# Dataset on the electronic and thermal transport properties of quaternary compounds of (PbTe)_0.95−x_(PbSe)_x_(PbS)_0.05_

**DOI:** 10.1016/j.dib.2017.05.041

**Published:** 2017-05-24

**Authors:** Dianta Ginting, Chan-Chieh Lin, Lydia Rathnam, Junpil Hwang, Woochul Kim, Rabih Al rahal Al Orabi, Jong-Soo Rhyee

**Affiliations:** a Department of Applied and Institute of Natural Science, Kyung Hee University, Yong-in, Gyeong-gi 17104, Republic of Korea; b Department of Mechanical Engineering, Yonsei University, Seoul 03722, Republic of Korea; c Department of Environmental Science and Engineering, Ewha Womans University, Seoul 03760, Republic of Korea

**Keywords:** Thermoelectric, PbTe, Thermal conductivity, Nano composite, Band convergence

## Abstract

The data presented in this article are related to the research article entitled “High thermoelectric performance in pseudo quaternary compounds of (PbTe)_0.95−*x*_(PbSe)_x_(PbS)_0.05_ by simultaneous band convergence and nano precipitation” (Ginting et al., 2017) [1]. We measured electrical and thermal transport properties such as temperature-dependent Hall carrier density *n*_*H*_, Hall mobility *μ*_*H*_, thermal diffusivity *D*, heat capacity *C*_*p*_, and power factor *S*^*2*^*σ* in (PbTe)_0.95−*x*_(PbSe)_x_(PbS)_0.05_ (*x*=0.0, 0.05, 0.10, 0.15, 0.20, 0.35, and 0.95) compounds with other related compounds from references. From the theoretical fitting of thermal conductivity *κ*, we found that the temperature-dependent thermal conductivity follows nano-structure model as well as alloy scattering. Transmission electron microscopy images shows that there are numerous nano-scale precipitates in a matrix. Owing to the low thermal conductivity and high power factor, we report high thermoelectric performances such as the high *ZT*, engineering *ZT*_*eng*_, efficiency *η*.

**Specifications Table**

**Table:** 

Subject area	Physics
More specific subject area	Materials
	Physics
Type of data	Table, image (TEM), text file, graph, figure
How data was acquired	TEM, Hall resistivity measurement (PPMS Dynacool 14T, Quantum Design, USA), Thermal diffusivity (LFA-447, NETZSCH, Germany)
Data format	Raw, Analyzed, Calculated
Experimental factors	TEM sample preparation: polish as the thin samples
	Hall resistivity measurement: polish as thin samples with rectangular shape and make a 5 point-contact via Pt or Au wire
	Thermal diffusivity: make circular plate sample (diameter 10 mm phi) with small thickness (<1 mm)
Experimental features	Electrical transport measurements provide Hall carrier density, Hall mobility, and power factor. Thermal transport measurements are thermal diffusivity and thermal conductivity. We compared the thermal conductivity with theoretical model fitting considering nano-structure and alloy scattering. Transmission electron microscope images show numerous nano-scale precipitation. We compare ZT values with other PbTe based compounds.
Data source location	Yong-In, Korea
Data accessibility	The data are available with this article. Some data for comparison are from references as indicated.

**Value of the data**

• The temperature-dependent Hall carrier density *n*_*H*_, Hall mobility *μ*_*H*_, thermal diffusivity *D* provides experimental understanding of the electrical and thermal transport properties on the compounds.
• Comparison of thermoelectric properties such as temperature-dependent power factor *S*^*2*^*σ*, *ZT* values, engineering *ZT* values *ZT*_*eng*_, and efficiency with other related compounds gives the level of the thermoelectric performance.
• Pisarenko plot of the Seebeck coefficient versus Hall carrier density shows that the compounds do not follow simple single parabolic band model.
• The additional electrical- and thermal-transport properties and their thermoelectric performance for the compounds have an importance in more profound analysis of the measurements.

## 1 Data

The Hall carrier density *n*_*H*_ and Hall mobility *μ*_*H*_ are obtained from the isothermal Hall resistivity *ρ*_*xy*_*(H)* and electrical resistivity *ρ* measurements using the relations of $R_{H}=\rho_{\mathrm{xy}}/H$, $n_{H}=-1/(R_{H}e)$, and $\mu_{H}=1/(\rho \mathrm{ne})$, respectively. Seebeck coefficient is measured by thermoelectric measurement system (ZEM-3, ULVAC, Japan). TEM images (High Resolution images/STEM/ED pattern) were collected using a JEOL 2100F at 200 kV. Energy dispersive x-ray spectrometer (EDS) analysis were obtained using Oxford Instruments (INCA platform) detector equipped on JEOM 2100F. Thermal diffusivity measurement is carried out by thermal conductivity measurement system (LFA-447, NETZSCH, Germany). Heat capacity is obtained from the Dulong-Petit fit using physical properties measurement system (PPMS Dynacool 14T, Quantum Design, USA).

## 2 Experimental design, materials and methods

The Table 1 presents the theoretical density, measured volumetric density, relative density, and specific heat of the compounds.

**Table 1:** Theoretical densities *D*_*T*_, measured volumetric densities *D*_*exp*_, relative densities *D*_*R*_, and specific heat *C*_*p*_ at room temperature of the (PbTe)_0.95−*x*_(PbSe)_x_(PbS)_0.05_ compounds.

*x*	*D_T_* (g cm^−3^)	*D_exp_* (g cm^−3^)	*D_R_* (%)	*C_p_* (J g^−1^ K^−1^)
0	8.13	7.88	96.92	0.156
0.05	8.12	8.02	98.70	0.157
0.1	8.10	7.92	97.70	0.158
0.15	8.08	7.90	98.01	0.160
0.2	8.06	7.88	97.76	0.161
0.35	8.14	7.98	98.03	0.165
0.95	8.17	8.00	97.91	0.182

The measured volumetric densities *D*_*exp*_ are all more than 96% of the theoretical densities *D*_*T*_, and the specific heat *C*_*p*_ is increased with the increase of Se concentration, which is calculated by using the equation *C*_*p*_/*k*_*B*_ per atom=3.07+4.7×10^−4^ (*T*/K−300) by fitting experimental data.

**Fig. 1:**
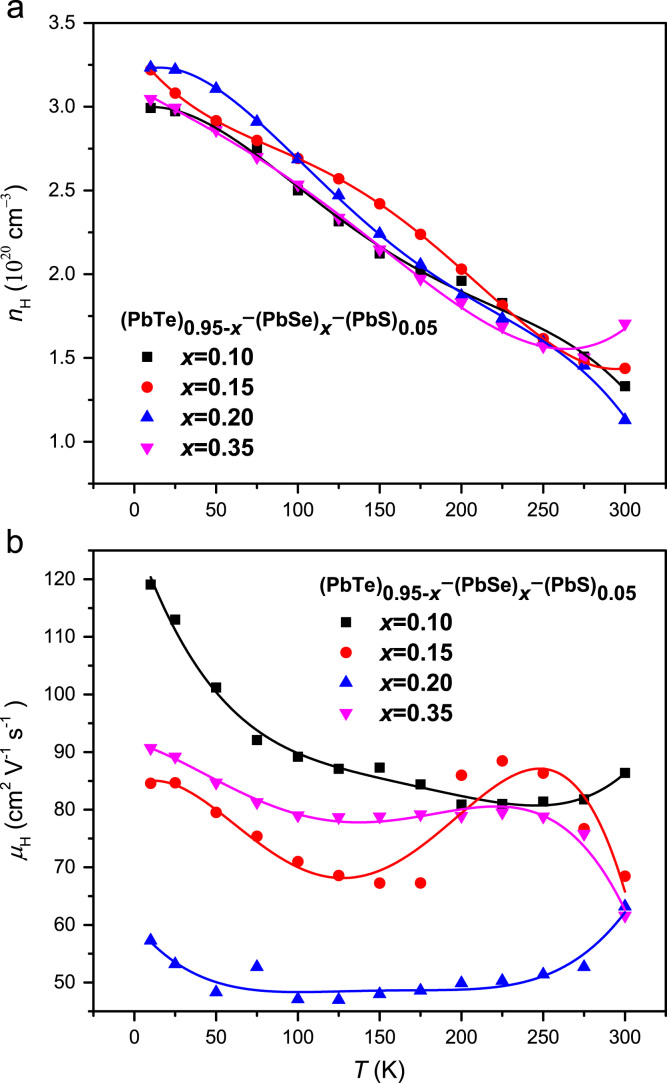
Temperature-dependent Hall carrier concentration *n*_*H*_ (a) and Hall mobility *μ*_*H*_ of (PbTe)_0.95−*x*_(PbSe)_x_(PbS)_0.05_ (*x*=0.1, 0.15, 0.2, and 0.35) (see the main article [1]).

Hall carrier concentrations *n*_*H*_ of the compounds are decreased with increasing temperature as shown in Fig. 1(a). The Hall carrier concentration is not sensitive with Se concentration in the (PbTe)_0.95−*x*_(PbSe)_x_(PbS)_0.05_ (*x*=0.1, 0.15, 0.2, and 0.35) compounds. Hall carrier mobilities are decreased with increasing Se concentration except *x*=0.35 case as presented in Fig. 1(b).

**Fig. 2:**
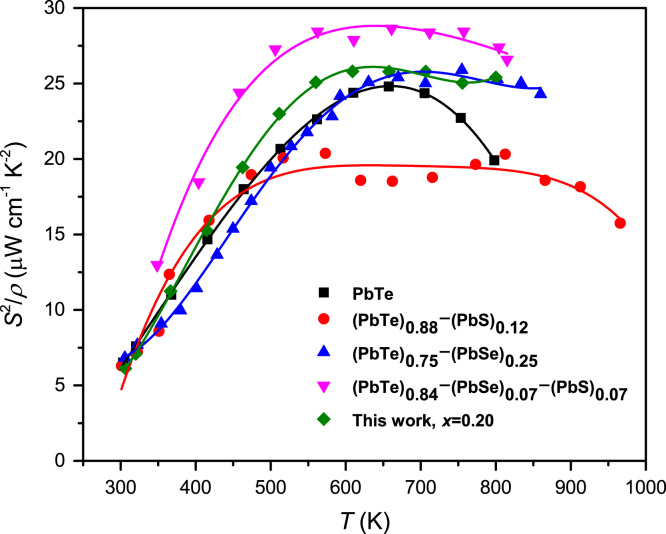
Comparison of power factors for (PbTe_0.95_Se_0.20_)(PbS)_0.05_ with (PbTe)_0.88_(PbS)_0.12_[2], (PbTe)_0.75_(PbSe)_0.25_[3], and (PbTe)_0.84_(PbSe)_0.07_(PbS)_0.07_[4] compounds.

The power factor *S*^*2*^*σ* of the pristine PbTe shows broad peak near 657 K for maximum value of 250 mW m^−1^ K^−2^, presented in Fig. 2. The (PbTe)_0.84_(PbSe)_0.07_(PbS)_0.07_ compound exhibits highest power factor 287 mW m^−1^ K^−2^. Our work for (PbTe)_0.95−*x*_(PbSe)_x_(PbS)_0.05_ (*x*=0.2, green diamond) is little bit decreased comparing with the state-of-the-art value of power factor.

**Fig. 3:**
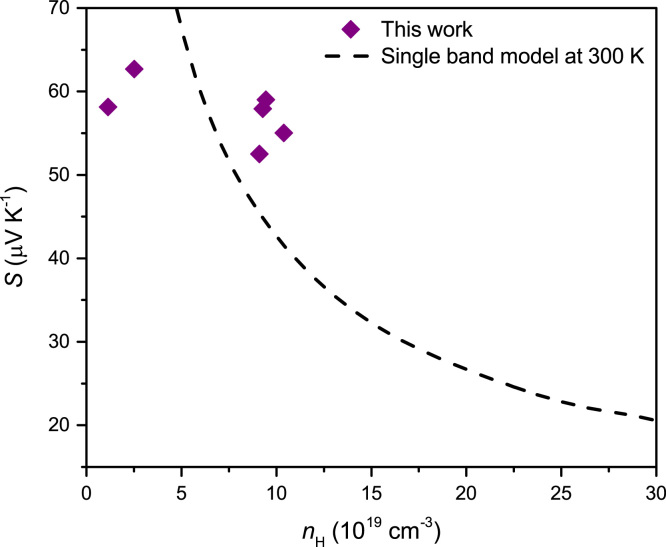
Room temperature Pisaranko plot based on single parabolic model (dashed line) with experimental data of the compounds.

The Pisaranko plot in Fig. 3 shows that the experiment results are deviated from the single parabolic model, indicating that the Seebeck coefficient is influenced by two band (light-band and heavy band) model.

**Fig. 4:**
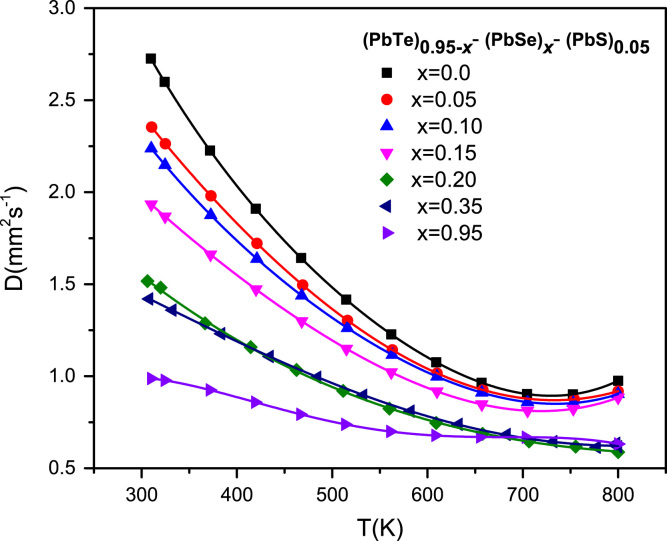
Temperature-dependent thermal diffusivities of the (PbTe)_0.95−*x*_(PbSe)_x_(PbS)_0.05_ compounds.

The thermal diffusivity in Fig. 4 is decreased with the increase of Se concentration. The thermal diffusivities are decreased with increasing temperature.

**Fig. 5:**
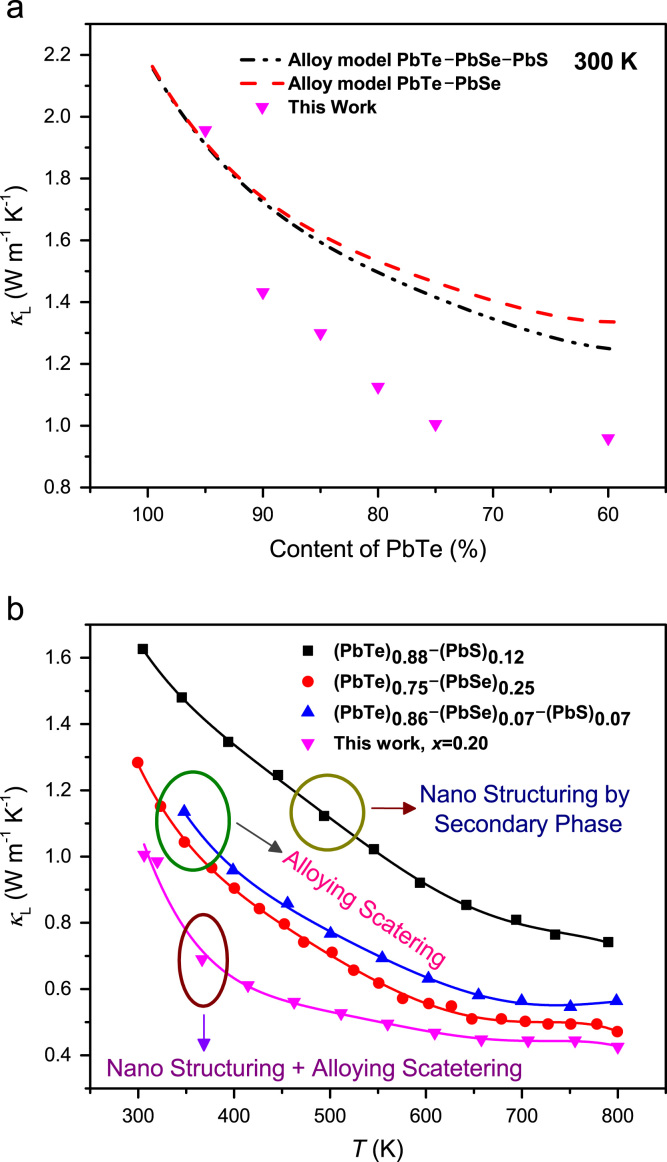
Comparison of theoretical lattice thermal conductivity for (PbTe)_1−x_(PbSe)_x_ and (PbTe)_1−x−y_(PbSe)_x_(PbS)_y_ alloys with respect to PbTe concentration base on Ref. [4]. (a) and temperature-dependent lattice thermal conductivity of (PbTe_0.95_Se_0.20_)(PbS)_0.05_ compound comparing with those of (PbTe)_0.88_(PbS)_0.12_[2]_,_ (PbTe)_0.75_(PbSe)_0.25_[3]_,_ and (PbTe)_0.86_(PbSe)_0.07_(PbS)_0.07_[4] compounds.

The lattice thermal conductivities, shown in Fig. 5(a), in this work are significantly lower than that calculated by using the alloy model of PbTe–PbSe–PbS and PbTe–PbSe, and it is also much reduced comparing with the previous reports by the nano-structuring as well as alloying scattering which is shown in Fig. 5(b).

**Fig. 6:**
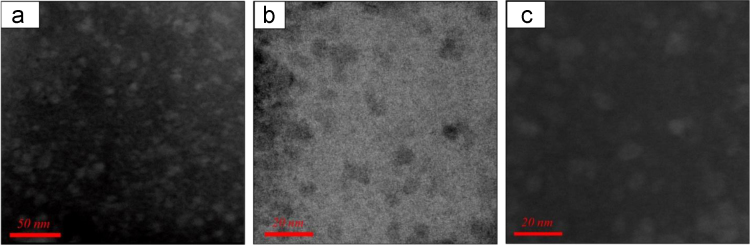
STEM images of (PbTe)_0.75_(PbSe)_0.20_(PbS)_0.05_: low magnification-high angle annular dark field (HAADF) image of numerous nano-precipitates with bright contrast (a), bright field (BF) (b), and HAADF images (c) with differences contrast of the same region.

Scanning Tunneling Electron Microscope (STEM) images in Fig. 6 show numerous nano-precipitations with the size of 5–10 nm inside the sample of (PbTe)_0.75_(PbSe)_0.20_(PbS)_0.05_.

**Fig. 7:**
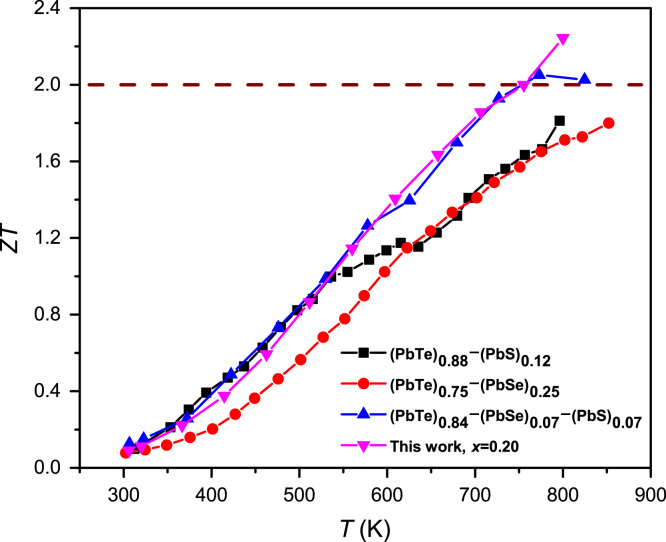
Dimensionless figure-of-merit *ZT* of (PbTe)_0.75_(PbSe)_0.20_(PbS)_0.05_ compounds comparing with (PbTe)_0.88_(PbS)_0.12_[2]_,_ (PbTe)_0.75_(PbSe)_0.25_[3]_,_ and (PbTe)_0.86_(PbSe)_0.07_(PbS)_0.07_[4].

Fig. 7 represents the thermoelectric figure-of-merit *ZT* values of the compounds and the other comparing materials as indicated from the references.

The sample of (PbTe)_0.75_(PbSe)_0.20_(PbS)_0.05_ compound obtains the highest *ZT* more than 2.2, which is higher than those of previously reported ones, as indicated from the references.

**Fig. 8:**
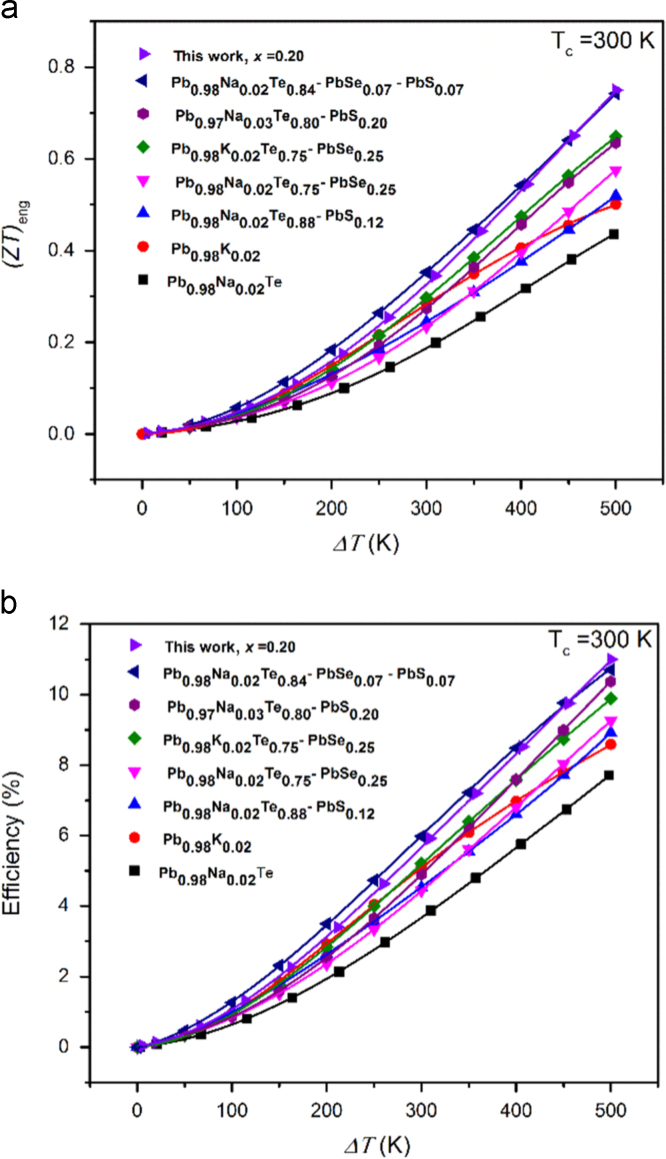
Comparative values of engineering *(ZT)*_*eng*_ (a) and efficiency *η* (b) in terms of temperature difference Δ*T* at *T*_*c*_=300 K for various compounds as indicated comparing with Pb_0.98_Na_0.02_Te, Pb_0.98_K_0.02_Te [5], (Pb_0.98_Na_0.02_Te_0.88_)(PbS)_0.12_[2], (Pb_0.98_Na_0.02_Te_0.75_)(PbSe)_0.25_[3], (Pb_0.98_Ka_0.02_Te_0.75_)(PbSe)_0.25_[5], (Pb_0.98_Na_0.02_Te_0.75_)(PbSe)_0.07_(PbS)_0.07_[4], (Pb_0.97_Na_0.03_Te_0.80_)(PbS)_0.20_[6].

Fig. 8 presents the comparative values of engineering *(ZT)_eng_* (a) and efficiency *η* (b) in terms of temperature difference *ΔT* at *T_c_* = 300 K for various compounds as indicated. The (PbTe)_0.75_(PbSe)_0.20_(PbS)_0.05_ sample shows both the highest (*ZT*)_eng_ of 0.75 and efficiency of 11% comparing with that of the previous reports.
